# Evolutionary aspects of resurrection ecology: Progress, scope, and applications—An overview

**DOI:** 10.1111/eva.12563

**Published:** 2017-11-22

**Authors:** Lawrence J. Weider, Punidan D. Jeyasingh, Dagmar Frisch

**Affiliations:** ^1^ Department of Biology Program in Ecology and Evolutionary Biology University of Oklahoma Norman OK USA; ^2^ Department of Integrative Biology Oklahoma State University Stillwater OK USA; ^3^ School of Biosciences University of Birmingham Birmingham UK

**Keywords:** dormant propagules, ecology, resurrection, seed/egg banks

## Abstract

This perspective provides an overview to the Special Issue on Resurrection Ecology (RE). It summarizes the contributions to this Special Issue, and provides background information and future prospects for the use of RE in both basic and applied evolutionary studies.

## 1 INTRODUCTION

Mutation, genetic drift, migration, and natural selection are processes that underlie phenotypic evolution (Fisher, 1930). Thus, predicting the evolution of any trait requires information on all of these processes, over at least a few generations. Such predictability is needed for applying evolutionary principles to solve problems in medicine, agriculture, biodiversity conservation, and environmental science. Note that the environment has a major bearing on particularly the last three of these processes, making predictions about trait evolution difficult (Endler, 1986). Currently, experimental evolution (i.e., “forward‐in‐time” method) is the most rigorous approach toward a quantitative understanding of trait evolution (Elena & Lenski, 2003). However, such studies are predominantly performed on microbes, or are limited to multicellular organisms with short generation times (although see Franks, Hamann, and Weis 2018, this issue, for another perspective). As such, experimental evolution is generally limited in assessing the evolution of complex traits, which could include pivotal trade‐offs in complex organisms with distinct life stages that express a variety of fitness‐relevant traits, and face a greater array of allocation decisions to maximize fitness. Further, such experiments are usually carried out in highly simplified ecological conditions that are unnatural in terms of both the abiotic and biotic niche parameters (Elena & Lenski, 2003).

The most common approach to studying natural populations is to substitute “space‐for‐time” to infer long‐term dynamics (Pickett, 1989). In other words, an investigator compares the population genetic parameters between two spatially separated populations differing in trait values to infer evolutionary mechanisms underlying trait divergence. Studies that use spatial comparisons to understand a temporal process assume that important events impacting trait evolution are impacted by spatial and temporal processes independently, but this assumption is rarely mentioned. Of course, this assumption is a necessity because even though paleo‐approaches allow us to measure trait values from preserved morphological or anatomical features (e.g., paleontology) and population genetic parameters (e.g., paleogenetics; Leonardi et al., 2017), we cannot go back in time to recover other critical traits related to biochemical, metabolic/physiological, behavioral, or life‐history mechanisms of organisms. Such a mechanistic understanding of complex traits is needed to refine evolutionary models to reach acceptable levels of predictability such that we can apply this knowledge to solve real‐world problems. How can we study the evolution of complex traits and entire phenomes in natural populations given insurmountable limitations of time travel?

## 2 OVERVIEW OF RESURRECTION ECOLOGY (RE): WHAT IS IT?

World cultures have been fascinated for centuries (if not millennia) about the concept of “time traveling,” particularly going back in time to a certain historical event or era. This continues to permeate modern pop culture around the world, due to motion pictures, books, television shows, among other forms of media. But what if the concept of time traveling to the past was actually more “science” than “science fiction”? The purpose of this special issue of *Evolutionary Applications* was to examine the burgeoning field of “resurrection ecology” (Kerfoot, Robbins, & Weider, 1999; Kerfoot & Weider, 2004; hereafter, abbreviated as RE), defined as the revival of long‐dormant organisms via hatching of dormant life stages such as seeds, eggs, and spores/cysts, thus enabling the direct quantification of phenotypes over time spans longer than the average human lifespan. Here, we examine how this relatively young field of study may help us better understand how organisms have adapted to variable historical or modern environmental challenges. For example, similar to understanding trait evolution by comparing extant populations that differ in trait values, one could examine trait evolution by taking a “paleo‐quantitative genetics/genomics” approach. This would consist of establishing crosses between modern‐era organisms with resurrected (“ancient”) organisms, which differ significantly in their mean values for the trait of interest. The resulting F1 hybrids could be selfed to produce a mapping population of F2 recombinant inbred lines (RIL). This F2 mapping population could then be subjected to a quantitative trait loci (QTL) analysis coupled with genome‐wide association studies (GWAS) using whole‐genome sequencing of the mapping population(s) and phenotypic assays. With a large enough mapping population (i.e., at least several hundred F2 RILs), this would allow one to identify genomic regions associated with the targeted trait. Such a resurrection ecology (RE) QTL approach would add greatly to our understanding of the underlying evolutionary trajectories of quantitative trait evolution in natural populations. Such a study using resurrected *Daphnia* clones is currently being conducted (R. Sherman, L. J. Weider and P. D. Jeyasingh, unpublished data). Along with a recent review article (Orsini et al., 2013), which covered much of the basics of RE, these combined approaches further highlight the potential utility of using RE to hind‐cast, in order to inform forecasting of evolutionary trajectories of organisms into the future.

In this special issue, we will follow up on certain aspects of this earlier coverage of RE (Orsini et al., 2013). We have gathered a series of papers from international experts in the field that address not only the ecological and evolutionary implications of RE, but also highlight the aspects applicable to some of the most pressing societal issues that humanity is facing (i.e., climate change, biodiversity loss, conservation, agriculture, and medical applications). The reader should note that this special issue will not be dealing extensively with the controversial concept of “de‐extinction,” at least in the narrow‐sense definition of this term (i.e., bringing extinct species back to life using genomics‐assisted methods; Shapiro, 2015, 2016). Rather, our focus will target organisms that produce long‐dormant (i.e., years to millennia) life stages that can be revived naturally; these include microbes, protists, plants, and a variety of invertebrate eukaryotes (Evans & Dennehy, 2005). From the perspective of researchers studying vertebrates, this might seem to be a taxonomically restricted assemblage of organisms. However, from a species/taxon‐level perspective, this group is not a trivial representation of the total biodiversity on our planet (Mora, Tittensor, Adl, Simpson, & Worm, 2011).

The use of these dormant propagules as a study system to look at temporal changes in genetic and ecological features of populations (and even communities) has been gaining considerable ground over the past 20–30 years (e.g., Cousyn et al., 2001; Decaestecker et al., 2007; Frisch et al., 2014, 2017; Geerts et al., 2015; Hairston & De Stasio, 1988; Hairston, Van Brunt, Kearns, & Engstrom, 1995; Hairston et al., 1999, 2001; Härnström, Ellegaard, Andersen, & Godhe, 2011; Kerfoot et al., 1999; Levin, 1990; McGraw, 1993; Rogalski, 2015, 2017; Vavrek, McGraw, & Bennington, 1991; Weider, Lampert, Wessels, Colbourne, & Limburg, 1997), building on earlier theoretical and empirical work on the evolutionary dynamics of seed banks (e.g., Templeton & Levin, 1979). We believe it is time to bring this emerging field of RE to a broader audience, which includes researchers, scientists, and general public stakeholders who are interested in (i) evolutionary adaptation to environmental change, comparing phenotypic and associated genetic and genomic changes of past and current populations; (ii) recovery of biodiversity using RE and restoration ecology—after both natural and anthropogenic environmental changes/stressors; (iii) the utility of archiving important “seed bank”/egg bank propagules (e.g., important crop plants, “heirloom” plants; germplasm/eggs) with applied aspects to agriculture or aquaculture (i.e., identifying agronomic genes related to such traits as seed dormancy—Denekamp et al., 2009; Prada, 2009); (iv) evolutionary medicine—studying “resurrected” microbes and their impacts on modern populations of humans and other species (e.g., the plague, anthrax, smallpox); (v) “dispersal from the past”—with climate/environmental change, how might “natural” dispersal from the past (e.g., melting of ice sheets/glaciers, thawing of permafrost, releasing long‐dormant cysts and propagules) impact evolutionary trajectories of modern populations.

## 3 RESURRECTION ECOLOGY (RE) APPROACHES

In addition to this overview manuscript, we received contributions from nine internationally recognized research groups. We tried to provide a balance among different organismal systems with representative contributions including aquatic invertebrates (i.e., *Daphnia*,* Artemia*), higher (i.e., terrestrial seed banks) and lower (i.e., phytoplankton cyst banks) plants, as well as microbial systems (i.e., microbes; pathogen–host systems). We have included both empirical studies and theoretical studies (i.e., Weis “invisible fraction bias”), and have asked a number of expert contributors to provide us with more specific reviews (e.g., paleolimnology—Burge, Edlund, and Frisch; dormancy as a life‐history strategy—Shoemaker and Lennon) on the current status and future direction of this burgeoning discipline.

For most of the short history of RE, a “back‐in‐time” approach has been taken (e.g., Kerfoot et al., 1999; Weider et al., 1997). This involves resuscitation of ancestral populations from either natural populations (e.g., collected from sediment cores) or archived populations (e.g., seed bank collections) and then comparing these ancestral lineages to modern‐day descendants. Many of the contributions to this special issue take a “back‐in‐time” approach and focus on specific model organisms (e.g., *Artemia*—Lenormand et al., 2018; bacteria—Houwenhuyse, Macke, Reyserhove, Bulteel, & Decaestecker, 2018; Shoemaker & Lennon, 2018; *Daphnia*—Goitom et al., 2018; Cuenca Cambronero, Bettina, & Orsini, 2018; phytoplankton—Ellegaard, Godhe, & Riberio, 2018). However, as pointed out in the contribution from Franks et al., 2018; this issue), a “forward‐in‐time” approach (a.k.a. “experimental evolution”) has been gaining momentum more recently (Elena & Lenski, 2003; Franks et al., 2008). This approach involves the purposeful establishment of an archived propagule bank (e.g., Project Baseline—Franks et al., 2008; Weis, 2018 this issue) that will serve as a research resource for the scientific community for at least decades into the future. Experimental evolution was pioneered by Richard Lenski and colleagues in their classic studies using the gut bacterium, *Escherichia coli*, as their model system (Elena & Lenski, 2003), where evolutionary forces (such as mutation and selection) and their impacts on trait evolution (e.g., resource utilization/growth rates) over the course of tens of thousands of generations (i.e., currently ~70,000 generations and counting) can be studied under controlled conditions. This “forward‐in‐time” approach has been expanded to eukaryotes, more specifically plants, which is highlighted in this special issue (Franks, Hamann and Weis, 2018; Weis, 2018). Our hope is that the reader can readily compare and contrast the utility of both of these RE approaches in studying both the basic and applied evolutionary dynamics of populations.

## 4 APPLIED EVOLUTIONARY ASPECTS OF RE: PROSPECTS AND LIMITATIONS

The invited contributors were asked to highlight the importance of RE in the study of evolutionary processes, and given the nature of this journal, to connect these approaches to more applied evolutionary challenges (see above). Burge, Edlund, and Frisch (2018) provide a comprehensive overview of the field of paleolimnology (i.e., the study of archived microfossils/biomarkers in the sediments of lakes/ponds), which has been critically important in reconstructing and understanding past climate and land‐use changes across millennial timescales. These authors highlight the importance of how these sediment archives also serve as natural repositories of dormant life stages of many organisms, and how “the marriage of necessity” between paleolimnology and RE can make critical contributions to our understanding of environmental/climate change/land‐use impacts on the evolutionary trajectories of aquatic organisms (e.g., phytoplankton—see Ellegaard et al., 2018 this issue; zooplankton—Frisch et al., 2014, 2017).

A key aspect of RE studies that can represent a significant challenge is the development of techniques for germinating/hatching/resuscitating dormant propagule life‐history stages. In this issue, Shoemaker and Lennon (2018) provide a review of dormancy as a critical life‐history feature of a wide variety of organisms, and look at how dormancy influences fundamental evolutionary forces at both the population genetic and macroevolutionary (i.e., speciation) levels. They focus primarily on microbial systems, but their population genetic model simulations are more broadly applicable to all organisms that use some form of dormancy as a life‐history strategy.

An important limitation with many RE studies that is highlighted by many authors in this special issue is the inverse relationship between germination/hatching success rate of dormant/diapausing propagules, and the length of time (i.e., age) that these dormant propagules have remained in the seed/egg/cyst bank. For the “RE poster child,” *Daphnia*, hatching success typically shows a precipitous drop from younger (i.e., from >75% for 20‐year‐old dormant eggs from sediments; Weider et al., 1997; Burge et al., 2018), to older propagules (i.e., ~0.03%—1% for centuries‐old propagules; Morton, Frisch, Jeyasingh, & Weider, 2015). Much of this reduction in hatching success is due to natural aging of these propagules in sediments that may be anoxic or even toxic (i.e., hydrogen sulfide—H_2_S; Weider et al., 1997). Thus, to this point in time, retrieving and reviving large numbers of propagules for most organisms (i.e., see exceptions for phytoplankton—Ellegaard et al., 2018 and microbes—Houwenhuyse et al., 2018; Shoemaker & Lennon, 2018) that date back much more than 70‐100 years remains challenging (Burge et al., 2018; Ellegaard et al., 2018; Frisch et al., 2014; Morton et al., 2015). Given the current rate of environmental change, and given what we know about contemporary evolution (e.g., Franks, Hamann, and Weis, 2018 this issue), even a 70‐ to 100‐year time span can represent enough spent generations (particularly, for short‐lived organisms), to allow sufficient time to track evolutionary changes, and thus, provide valuable insights into the evolutionary processes in both pristine and human‐impacted populations.

Another potential limitation of RE in reconstructing evolutionary trajectories for different traits is highlighted by Weis (2018, this issue), who models what is termed the “invisible fraction.” This phenomenon can occur *“if seed (propagule) traits that affect survival during storage (dormancy) and revival are genetically correlated to adult traits of interest”* (Weis, 2018). In other words when using “back‐in‐time” or “forward‐in‐time” RE approaches, one needs to be concerned with whether the measurement of the phenotypic traits of interest in the resurrected individuals is truly representative of the entire population or whether it may be biased by any differential hatching/germination success. This could become a more acute issue, when revival success rates are low. Weis (2018) indicates that this effect may be trivial in certain cases; or alternatively, significant bias may be present. The intensity of this bias will depend on whether selection is operating extensively on traits that affect seed survival, and also, the strength of genetic correlations between these seed (survival) traits and the adult traits of interest. He suggests that one way to reduce this bias (at least for “forward‐in‐time” RE studies) is to have a well‐structured pedigree (i.e., family structure) available in order to test correlations between the family means among these traits. With further development of new statistical methods (see Weis, 2018) that incorporate pedigree/genealogical data, this bias may be correctable. This bias becomes less severe if one is dealing with resurrecting asexual (clonal) lineages, where genealogical ancestry is more certain, and where only mutational input might be an issue depending on the number of generations that propagules have remained sequestered in the seed/egg bank.

In addition, previous RE work using the *Daphnia* system (Weider et al., 1997; Jankowski & Straile, 2003; L. J. Weider, unpublished data) has shown that in general, very little bias has been observed in the genetic (genotypic) composition of the hatching fraction of the populations versus the unhatched portions, at least dating back ~40–50 years. Clearly, as we go deeper in time in a propagule bank, with the aforementioned issues related to reduced hatching success and smaller hatching fractions, the potential for bias increases.

Other applied aspects of RE that are represented by contributions to this special issue, and that are important in understanding the ecology and evolution of natural populations and communities include (i) invasive species biology (i.e., *Artemia*—Lenormand et al., 2018); (ii) the role of RE in possible pathogen–host interactions that impact both human and nonhuman populations via “dispersal from the past” (i.e., melting permafrost releasing microbial pathogens—Houwenhuyse et al., 2018); (iii) climate and land‐use changes impacting nutrient enrichment (i.e., eutrophication of aquatic systems—Ellegaard et al., 2018; Cuenca Cambronero et al., 2018); and (iv) evolutionary feedback and ecosystem functioning (i.e., Goitom et al., 2018).

As mentioned above, experimental evolution studies that were pioneered using the *E. coli* system (Elena & Lenski, 2003) can now be examined in both “forward‐in‐time” (Franks et al., 2018; Weis, 2018) and “back‐in‐time” RE studies. Further, a couple of experimental studies that use the latter approach focus on the *Daphnia* model system in this special issue. Cuenca Cambronero et al. (2018) demonstrated that over the course of 50 years, they could track the impacts of nutrient pollution (i.e., eutrophication) mediated by increasing temperatures (and decreasing oxygen) in aquatic systems. This resulted in differential competitive success among resurrected genotypes of *Daphnia magna* from different time periods that varied in hemoglobin (Hb) production under nonstressed (normal) and stressed (elevated) temperatures. Both genetic and plastic responses were observed, and the authors go on to suggest that impacted waterbodies may benefit from using translocated “winner” genotypes, as a strategy to avoid local population extirpations under current increasing thermal (and nutrient) environments.

In another experimental *Daphnia* study, Goitom et al. (2018) used resurrected *D. magna* genotypes from three different time periods from a pond that historically varied in the intensity of fish predation (i.e., time span ~20 years). In experimental mesocosms, these authors tracked subpopulation (i.e., genotypic) differences in population dynamics (i.e., densities and relative ratios of juveniles to adults) in the presence/absence of fish predators. Results revealed differences in population dynamics and top‐down control of primary productivity (i.e., algal production) between mesocosms harboring the different resurrected subpopulations. They observed an evo‐eco feedback that demonstrated trophic‐level and ecosystem processes can be impacted by rapid evolution in grazer (i.e., *Daphnia*) populations to changes in predation pressure (by fish). These authors suggest an important applied role of resurrection ecology for demonstrating the effect of rapid evolution that can lead to alterations at the ecosystem level. Indeed, Roy Chowdhury and Jeyasingh (2016) found that differential phosphorus recycling of ancient and extant *Daphnia* clones impacted algal stoichiometry and abundance, with potentially important implications for the structure and functioning of lake ecosystems that they inhabit.

Additional applied evolutionary aspects include the study of invasive species. This is highlighted by Lenormand et al. (2018, this issue), who used RE approaches to study the invasion of salterns in southern France by the invasive brine shrimp, *Artemia franciscana*. Humans have served as the purposeful vector for this species, and have spread it from its native range in North America to every continent (except Antarctica). Like all *Artemia* species, it produces a highly resistant encapsulated diapausing/dormant cyst that rests in a sediment bank and can withstand extreme fluctuations in environmental conditions (i.e., temperature, salinity). It is this life‐history stage that is harvested in massive quantities (Lenormand et al., 2018), making it a species of significant commercial importance in the aquaculture trade (i.e., as food for fish culturing). Lenormand and colleagues present a case study of *A. franciscana* invasion into a saltern in southern France that is inhabited by the native *A. parthenogenetica* and use RE approaches to document the invasion process. This example is reminiscent of previous studies that used paleolimnological techniques to track the invasion dynamics of *Daphnia* species/clones, both locally (e.g., Duffy, Perry, Kearns, Weider, & Hairston, 2000), and on a continent‐wide scale (e.g., Mergeay, Vanoverbeke, Verschuren, & De Meester, 2007; Mergeay, Verschuren, & De Meester, 2006).

Another contribution by Houwenhuyse et al. (2018) provides a review of the potential “dispersal from the past” of potential pathogens, and how re‐emergence of ancient microbes and viruses may pose risks to modern‐day hosts. Recent examples of the release of active pathogens from Arctic permafrost (e.g., anthrax from 75‐year‐old reindeer carcasses in Russia) due to climate change raise an important applied aspect of RE related to human health and nonhuman disease epidemiology. Additional evidence of more ancient “resurrections” from microbes dating back into the early or late Pleistocene (e.g., Bidle, Lee, Marchant, & Falkowski, 2007; Legendre et al., 2014) also adds a dimension of how these ancient lineages may alter the evolutionary trajectories of modern‐day populations. Resurrection ecology has much to add to tracking such “dispersal‐from‐the‐past” events.

## 5 FUTURE DIRECTIONS: WHERE DO WE GO FROM HERE?

### 5.1 What new tools/techniques/approaches can be used and how does their application teach us something about evolution that modern organisms cannot?

Identification of genes and molecules that play important roles in adaptation to environmental change or that are involved in the evolution of pathogen resistance allows important discoveries with the potential to provide practical benefits in applied fields such as environmental and human health (e.g., Ledford, 2017). However, genomic resources alone that may be available from whole‐genome sequencing of ancient isolates cannot provide the key information necessary to discover genomic adaptation that translates to phenotypic change and associated downstream processes including gene regulation, gene expression, translation, and a diversity of cellular processes.

Resurrection ecology (RE) provides science with living historic organisms (as opposed to, e.g., museum specimens or fossil DNA) that can be raised in laboratory or semi‐laboratory (e.g., experimental plots) conditions in order to characterize phenotypic evolution and the underlying genetic architecture, using temporal snapshots of the same population with the same genetic background. For example, new developments in paleoecology and paleolimnology (see Burge et al., 2018) highlight advancements in new methodologies for dating and environmental reconstruction (e.g., multiproxy biochemical and molecular assays) that, when integrated with RE and other (genomic) techniques, should provide powerful new approaches for advancing this emerging field.

Controlled experiments link these resurrected phenotypes with the same technologies that are available and applicable to modern organisms (e.g., phytoplankton—see Ellegaard et al., 2018—this issue). For example, the various “‐omics” fields (e.g., transcriptomics, metabolomics, proteomics) and gene‐editing technologies (e.g., CRISPR‐Cas9; Jinek et al., 2012) are applicable in the same way as they are to extant organisms. Note: such gene‐editing technologies are not without controversy (e.g., Hampton, 2016) or limitations (e.g., see Drury, Draper, Siniard, Zentner, & Wade, 2017 for limitations of this technique in natural populations).

Currently available case studies of such research are scarce, and predominantly include work on the *Daphnia* model and the comparison of isolates that were resurrected from times predating environmental disturbance. In particular, research has focused on genome‐wide association studies (GWAS; e.g., Orsini, Spanier, & De Meester, 2012) and the transcriptomic responses of historic isolates resurrected from time periods predating human disturbance and their comparison to modern isolates (Roy Chowdhury et al., 2015).

In future work, whole‐genome bisulfite sequencing (WGBS) can be applied to compare the methylome of resurrected and extant isolates to uncover evolutionary changes at the epigenetic level. Akin to the synthetic resurrection of ancestral proviral proteins that have the potential to provide resistance of agricultural plants to contemporary viruses (Delgado, Arco, Ibarra‐Molero, & Sanchez‐Ruiz, 2017), or the wedding of synthetic biology with ancient sequence reconstruction for experimental evolution studies with *E. coli* (Kaçar & Gaucher, 2012; also see Houwenhuyse et al., 2018, this issue), ancient biomolecules can be isolated from resurrected organisms and inserted into modern organisms using gene engineering technologies such as the CRISPR‐Cas9 system (Jinek et al., 2012).

These emerging technologies have been a centerpiece for one topic—”de‐extinction” (Shapiro, 2015), which has certainly been making headlines related to the sensational possibilities of bringing long‐extinct organisms like the woolly mammoth back to life. The use of some of the molecular techniques (i.e., cloning, gene‐editing) mentioned here is pushing these efforts forward; however, controversy (Cohen, 2014) and technological limitations are apparent (Shapiro, 2016). As pointed out in a recent review of de‐extinction technologies, Shapiro (2016) indicated that even with the use of cloning (e.g., somatic cell nuclear transfer), gene‐editing, and/or “back‐breeding” (i.e., selective breeding), these efforts will not result in the de‐extinction of the actual organism, but rather, “an ecological proxy”. We will leave it up to the reader to decide for themselves, whether the practical limitations and ethical aspects of “de‐extinction,” as cited here, are an insurmountable challenge to further development of this research field.

Additional future research using results from RE studies may help us delve into gene discovery related to important applied life‐history features such as dormancy, which may have important applications from the perspective of aquaculture (e.g., Denekamp et al., 2009) and/or agriculture (e.g., Bentsink et al., 2010; Prada, 2009). Might the study of dormancy‐related traits (see Shoemaker & Lennon, 2018) via resurrection ecology (RE) studies also have more futuristic applications? Might research in cryopreservation (e.g., gamete or zygote preservation for animal husbandry, conservation biology, or medical applications; Holt & Pickard, 1999) benefit from RE studies, particularly related to long‐term preservation of propagules in ice cores/permafrost (Bidle et al., 2007; Houwenhuyse et al., 2018; Shoemaker & Lennon, 2018; Yashina et al., 2012)? Is there a role for resurrection ecology in the emerging field of restoration ecology related to the recovery and re‐establishment of extirpated populations or species on both a local (Uesugi, Nishihiro, Tsumura, & Washitani, 2007) and landscape scale (Merritt & Dixon, 2011)? Finally, might future long‐term extraplanetary exploration (colonization?) benefit from background RE research conducted on the breaking of dormancy for the long‐term transport of seeds or diapausing propagules (e.g., Alekseev, Hwang, & Stseng, 2006)?

## 6 SUMMARY/CONCLUSIONS

In conclusion, we hope that the collection of contributions presented in this special issue on basic and applied evolutionary aspects of RE will stimulate the reader to delve more deeply into this emerging field. From a population‐level perspective, organisms may evolve a multitude of strategies to deal with environmental challenges, many of which would be overlooked in a laboratory setting purely concerned with experimental evolution, where selective regimes are highly controlled and divergent phenotypes are the exception (Bailey & Bataillon, 2016). In contrast, by allowing one to track evolutionary trajectories in the natural environment under similar or parallel, but more complex selective scenarios for multiple, unrelated populations, RE has an important role to play in evolutionary studies.

Of course, RE is not a panacea for studying evolution. As we point out in this overview article, and as the reader can see in a number of contributions to this special issue, there are certain limitations in using this approach (e.g., not all taxonomic groups render themselves to such studies; possible “hatching/germination bias”). However, given the complexities of studying evolutionary processes in natural populations, especially in light of traditional “space‐for‐time” limitations (Pickett, 1989), we see RE as providing clear benefits. The main benefit is that one can actually revive not only “whole genomes” but also “whole phenomes,” and begin to more fully examine complex trait evolution over timescales that exceed the typical lifespan of a research project and/or investigator. Although the potential for RE to reveal the diversity of adaptive evolution is only in its infancy, we contend that much remains to be learned from this unique approach. Expanding RE to include other organisms suitable for this approach, and mitigating key inherent limitations as this collection of papers do, is an ideal and much‐needed complement to mainstream studies on phenotypic evolution, which substitute space for time to infer adaptive (temporal) trait dynamics.

Finally, as pointed out throughout this special issue, aspects of RE can impact a number of critically important applied disciplines, including agriculture (e.g., seed dormancy), biomedicine (e.g., pathogen release), biodiversity conservation, and ecosystem restoration (e.g., “dispersal from the past”). The use of RE in more applied studies may prove valuable in helping to solve some of the most pressing societal/environmental challenges that we face as a species (Carroll et al., 2014).
